# Prevalence, Incidence and Associates of Pulmonary Hypertension Complicating Type 2 Diabetes: Insights from the Fremantle Diabetes Study Phase 2 and National Echocardiographic Database of Australia

**DOI:** 10.3390/jcm10194503

**Published:** 2021-09-29

**Authors:** Nishant Nundlall, David Playford, Geoff Strange, Timothy M. E. Davis, Wendy A. Davis

**Affiliations:** 1 School of Medicine, The University of Notre Dame, Fremantle, WA 6160, Australia; nishantnundlall@gmail.com (N.N.); david@playford.biz (D.P.); geoff@mozaicsolutions.com.au (G.S.); 2 The Heart Research Institute, Newtown, NSW 2042, Australia; 3 Department of Cardiology, Royal Prince Alfred Hospital, Camperdown, NSW 2050, Australia; 4 Sydney Medical School, Faculty of Medicine and Health, The University of Sydney, Sydney, NSW 2006, Australia; 5 Fremantle Hospital, Medical School, The University of Western Australia, Fremantle, WA 6160, Australia; wendy.davis@uwa.edu.au

**Keywords:** type 2 diabetes, pulmonary hypertension, prevalence, incidence, risk factors

## Abstract

There is a paucity of epidemiologic data examining the relationship between pulmonary hypertension (PH) and diabetes. The aim of this study was to determine prevalence, incidence and associates of PH complicating type 2 diabetes. Data from 1430 participants (mean age 65.5 years, 51.5% males) in the Fremantle Diabetes Study Phase 2 (FDS2) were linked with the National Echocardiographic Database of Australia (NEDA) to ascertain the prevalence and incidence of PH (estimated right ventricular systolic pressure (eRVSP) >30 mmHg as a new suggested threshold or the conventional >40 mmHg) over a 12-year period. PH prevalence in FDS2 was compared with that in NEDA overall and a geographically close sub-population. Multivariable analyses identified associates of prevalent/incident PH in the FDS2 cohort. Of 275 FDS2 patients (19.2%) with pre-entry echocardiography, 90 had eRVSP >30 mmHg and 35 had eRVSP >40 mmHg (prevalences 32.7% (95% CI 27.3–38.7%) and 12.7% (9.1–17.4%), respectively), rates that are 35–50% greater than national/local NEDA general population estimates. Moreover, 70 (5.0%) and 123 (9.2%) FDS2 participants were identified with incident PH at the respective eRVSP thresholds (incidence (95% CI) 7.6 (6.0–9.7) and 14.2 (11.8–17.0)/1000 person-years), paralleling data from recognised high-risk conditions such as systemic sclerosis. The baseline plasma N-terminal pro-brain natriuretic peptide concentration was the strongest independent associate of prevalent/incident PH. Approximately 1 in 8 people with type 2 diabetes have PH using the eRVSP >40 mmHg threshold. Its presence should be considered as part of regular clinical assessment of individuals with type 2 diabetes.

## 1. Introduction

Pulmonary hypertension (PH) is a condition with significant associated morbidity and mortality [1]. Its diagnosis requires measurement of the mean pulmonary artery pressure (mPAP) during right heart catheterization (RHC), with classification into one of five sub-groups based on additional clinical, pathophysiologic and hemodynamic features [2]. Doppler echocardiography has, however, become the first line investigation where PH is suspected because it provides a non-invasive surrogate of mPAP through an estimate of right ventricular systolic pressure (eRVSP) [3]. The prevalence of PH detected in this way in recent general population studies has ranged between 2.6% and 9.4% based on an eRVSP >40 mmHg [4,5,6,7,8]. The variation in these figures may reflect differences in data sources, from population-based individual participant screening [4,6] to the interrogation of echocardiographic databases derived from investigation of possible or pre-existing cardiac disease [5,7,8]. Notwithstanding these differences, the burden of PH in the community appears significant, and there is evidence that a lower eRVSP threshold of >30 mmHg may be more appropriate than the conventional >40 mmHg as a predictor of increased mortality [8,9].

Preclinical data suggest that diabetes increases the risk of PH [10,11] but epidemiologic studies show inconsistent results. In relatively small-scale general population echocardiographic screening studies, diabetes was an independent associate of prevalent PH in African Americans [4] but not in a European population [6]. Regarding PH sub-types, diabetes was a significant independent risk factor for the prevalence of Group 1 PH (pulmonary arterial hypertension (PAH)) ascertained from a large US administrative database [12] but not for PAH incidence in the smaller community-based Fremantle Diabetes Study (FDS) Phase I cohort [13]. Diabetes should increase the risk of Group 2 PH because of the associated increase in left heart disease [14], but there are no published supportive data. There is, however, evidence that diabetes is associated with Group 3 PH, which is due to lung disease/hypoxia [15].

There have been no studies of the frequency and associates of PH in well-characterized cohorts of people with type 2 diabetes. Such studies would allow comparison with contemporary general population data collected in the same way, as well as providing evidence of diabetes-specific etiologic factors for PH. We have, therefore, utilized detailed longitudinal data from the FDS Phase II (FDS2) and linked data from a large national echocardiographic database to investigate the prevalence, incidence and determinants of PH in representative community-based individuals with type 2 diabetes.

## 2. Materials and Methods

### 2.1. Study Site, Participants and Approvals

**Figure 1:**
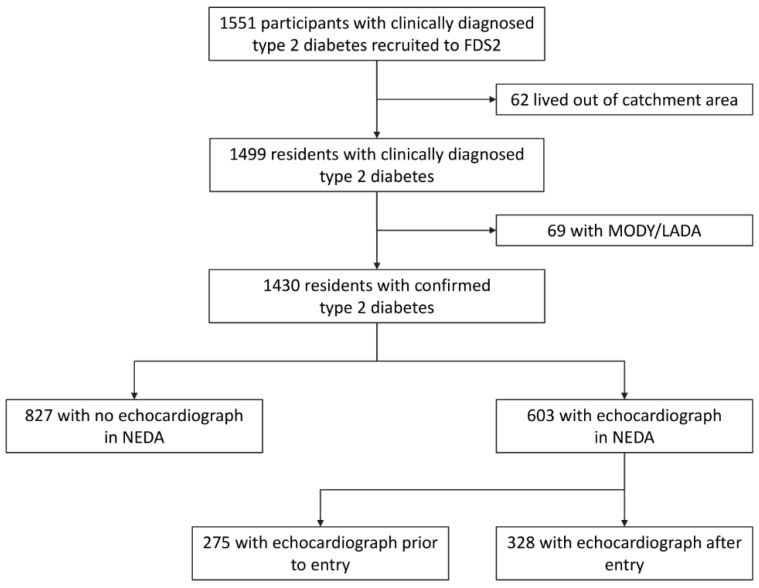
Consort diagram showing the number of Fremantle Diabetes Study Phase 2 participants included in the present sub-study.

The FDS2 is a longitudinal observational cohort study of residents with diabetes recruited from a zip code-defined urban community of 157,000 people in the state of Western Australia (WA). The FDS2 sample characteristics, including classification of diabetes and details of non-recruited participants, have been described previously [16]. Of 4639 eligible residents identified, 1668 (36%) were recruited to the FDS2. Of these, 1499 (89.9%) had clinically-defined type 2 diabetes, but after genetic/serologic screening for Maturity Onset Diabetes of the Young and Latent Autoimmune Diabetes of Adults, 1430 with confirmed type 2 diabetes were included (Figure 1). The South Metropolitan Area Health Service Human Research Ethics Committee approved FDS2. Written informed consent was obtained from each participant.

### 2.2. Clinical Assessment

Each FDS2 participant was comprehensively assessed at entry between 2008 and 2011 and was invited to subsequent biennial reviews. Assessments comprised questionnaires covering socioeconomic, demographic and lifestyle data, health care utilization, all medical conditions and medication use. A physical examination was conducted by trained nurses according to a standard protocol. Biochemical tests were performed on fasting samples using validated automated methods in a single nationally accredited laboratory. Complications were identified using standard definitions [17].

### 2.3. Echocardiography Database and Parameters

The National Echocardiographic Database of Australia (NEDA; Australian New Zealand Clinical Trials Registry ACTRN12617001387314) is an observational registry that captures individual echocardiographic and demographic data on a retrospective and prospective basis from participating Australian centers [18]. Approval was obtained from all relevant research ethics committees in each Australian state and territory. As of January 2020, 28 centers had contributed >1000,000 investigations (with nearly 50 million individual measurements) from >600,000 individuals. All data in each center’s echocardiography database are remotely transferred to a central database using vendor-agnostic, automated data extraction. Precise definitions for each echocardiographic variable are applied. Units are transformed to the NEDA standard, and repeated measures for the same variable are converted to a single value [18]. The eRVSP is derived using the Bernoulli equation (RVSP = TRV^2^ + 5 mmHg) where TRV represents the peak tricuspid regurgitation velocity (TRV) and 5 mmHg represents a conservative estimate of the right atrial pressure. Where no tricuspid regurgitation (TR) was present or the TR jet was insufficient to measure a peak velocity, the TRV is assumed normal and PH presumed absent. Since echocardiography was performed for clinical indications such as dyspnea, PH was assumed absent in participants who did not undergo echocardiography. All-cause PH was examined without attributing etiology or PH classification.

### 2.4. Data Linkage

Linkage of the NEDA and FDS2 databases was performed in a secure data warehouse under ethical approval from the University of Notre Dame Human Research Ethics Committee. There were 353,093 echocardiographs from FDS2 postcodes in the NEDA database. Matching of FDS2 participants to echocardiographs was by sex, surname, first name, date of birth and date of death (when applicable). Possible matches were cross-checked with height and weight from the FDS2 and NEDA databases, and, where a death was recorded after 2016, death dates were validated using the Perth Metropolitan Cemeteries Board database. This procedure resulted in 702 matches (40.5% of the whole FDS2 cohort).

### 2.5. Ascertainment of Co-Morbidities

Access to WA morbidity/mortality data for FDS2 participants continues through the WA Data Linkage System (WADLS) [19] as approved by the WA Department of Health Human Research Ethics Committee. All public/private hospitalizations in WA are recorded in the Hospital Morbidity Data Collection (HMDC) established in 1970. The latest linkage has data to end-2016. The HMDC was used to verify data relating to complications and co-morbidities obtained in FDS2 assessment and to the Charlson Co-morbidity Index (CCI) in the previous 5 years excluding diabetes and its complications [20].

Linkage with WA hospital morbidity data from July 1999 (when International Classification of Diseases (ICD) 10-AM coding was introduced) using diagnosis codes I27.0 and I27.2 provided prevalent and incident hospitalizations for/with PH, which were used for validation purposes. Two participants without PH identified from echocardiography had a hospitalization for/with PH before entry and, to be conservative, were excluded from the estimates of PH prevalence and incidence. One of these cases was hospitalized prior to inception of NEDA and was subsequently captured by NEDA during follow-up and the other was not captured by NEDA but was rehospitalized with PH during follow-up.

### 2.6. Statistical Analysis

The computer packages IBM SPSS Statistics 25 (IBM Corporation, Armonk, NY, USA) and StataSE 15 (College Station, TX: StataCorp LP) were used for statistical analysis. Data are presented as percentages, mean ± SD, geometric mean (SD range) or, in the case of variables which did not conform to a normal or log-normal distribution, median and inter-quartile range (IQR). Two-sample comparisons were by Fisher’s exact test, Student’s *t*-test or Mann–Whitney U-test as appropriate. Prevalent PH was taken as that on echocardiography between 2004 and study entry. Incident PH was determined using echocardiography between entry and end of 2016, with prevalent cases at baseline excluded.

For the whole FDS2 cohort, multivariable analyses were performed for each eRVSP threshold (>30 and >40 mmHg) using datasets that included multiply imputed (×20) variables for those with missing values (up to 1.7% of participants). Multiple logistic regression and Cox proportional hazards modelling were used to identify baseline associates of prevalent and incident PH, respectively, from clinically plausible baseline variables with *p* < 0.20 in bivariable analyses. Fine and Gray modelling was used to assess the influence of the competing risk of death [21]. The proportional hazards assumption was checked for each model using time-varying covariates. When this assumption was violated, a time-varying interaction of the covariate with ln(time) was included in the model.

## 3. Results

### 3.1. Baseline Characteristics

The mean ± SD age of the 1430 participants was 65.5 ± 11.6 years at entry, 51.5% were males and their body mass index (BMI) was 31.3 ± 6.1 kg/m^2^. Their median {IQR} diabetes duration was 8.0 {2.5–15.4} years, their median HbA_1c_ 6.8 {6.2–7.7}% (51 {44–61} mmol/mol) and 15.7 % were insulin-treated. The characteristics of participants in the cohort who were and were not captured by the NEDA database (42.2 % and 57.8%, respectively; Figure 1) are shown in Supplementary Table S1 and the associated odds ratios in Supplementary Table S2. Compared to those without a documented echocardiograph, those with a NEDA record were younger at diabetes diagnosis and who had a higher BMI and lower estimated glomerular filtration rate (eGFR) were more likely to have a history of coronary heart disease and to be taking aspirin, to have a higher plasma N-terminal pro-brain natriuretic peptide (NT-proBNP) concentration and to have a greater comorbidity burden. Similarly, the characteristics of participants captured by NEDA who did not have or who had a measurable/measured eRVSP (40.1 % and 59.9%, respectively; Figure 1) are shown in Supplementary Table S1. Compared to those without eRVSP data, participants with a valid eRVSP were older, more likely female, leaner, less likely to be insulin-treated and more likely to have renal impairment, atrial fibrillation and heart failure (Supplementary Table S2).

### 3.2. Prevalence and Associates of Pulmonary Hypertension

The baseline characteristics of participants compared by prevalent PH status for the two eRVSP thresholds are summarized in Table 1. Of the 275 patients (19.2%) with at least 1 echocardiogram prior to study entry, 90 had an eRVSP >30 mmHg and 35 had an eRVSP >40 mmHg. The respective prevalences were 32.7% (95% CI 27.3–38.7%) and 12.7% (9.1–17.4%). If the denominator is restricted to the 168 with an echocardiogram and a valid eRVSP measurement, the respective prevalences were 53.6% (45.7, 61.2%) and 20.8% (15.1, 27.9%). Under the most conservative scenario, that is, using the total 1430 FDS2 participants as the denominator, the minimum indicative prevalences of echocardiographically identified PH at eRVSPs >30 mmHg and >40 mmHg were 6.3% (5.1–7.7%) and 2.5% (1.7–3.4%), respectively. Including the two participants with hospitalizations for/with PH but no NEDA identified PH prior to study entry, the respective minimum indicative prevalences would be slightly higher at 6.4% (5.2, 7.9%) and 2.6% (1.9, 3.6%).

The 92 and 37 participants identified with PH at these thresholds were more likely to be older, have longer diabetes duration, have higher a body shape index (ABSI) as a marker of visceral obesity [22], be taking antihypertensive medications and have lower serum total cholesterol than the remaining 1338 and 1393 participants, respectively, with confirmed type 2 diabetes. Those with prevalent PH also had more atrial fibrillation, cerebrovascular disease, heart failure, higher NT-proBNP, peripheral sensory neuropathy, cardiac valvular disease, history of chronic obstructive pulmonary disease (COPD) and obstructive sleep apnea. The independent associates of prevalent PH under each eRVSP threshold are shown in Table 2. Prevalent PH at eRVSP >40 mmHg was associated with a history of heart failure and COPD and higher levels of NT-proBNP. Prevalent PH at eRVSP >30 mmHg was associated with a history of valvular disease and heart failure, aspirin use, orthostatic hypotension and higher levels of NT-proBNP.

### 3.3. Incidence of Pulmonary Hypertension and Its Predictors

During 9162 person-years (6.6 ± 1.9 years) of follow-up, 70 participants (5.0%; incident rate (IR) (95% CI) 7.6 (6.0, 9.7)/1000 person-years) were identified with an incident PH at eRVSP >40 mmHg. During 8660 person-years (mean 6.5 ± 2.0 years) of follow-up, 123 participants were identified with incident PH based on an eRVSP >30 mmHg (9.2%; 14.2 (11.8, 17.0)/1000 person-years). In bivariable analyses (Table 3), the participants with incident PH at both thresholds of eRVSP were more likely to be older, have longer duration of diabetes, be treated for hypertension and to be taking aspirin. The serum high-sensitivity C-reactive protein (hsCRP) and plasma NTpro–BNP concentrations were also higher. Participants with incident PH were more likely to have atrial fibrillation, coronary artery disease, peripheral artery disease, cerebrovascular disease, a history of heart failure and a higher urinary albumin:creatinine ratio.

The independent baseline predictors of incident PH are shown in Table 2. For an eRVSP >40 mmHg, these comprised older age, being married/*de facto* (protective), increasing diabetes duration and heart rate, lipid-lowering medications and both hsCRP and NT-proBNP. For RVSP >30 mmHg, the independent predictors were older age, male sex (protective), increasing diabetes duration and hsCRP and NT-proBNP. In general, HRs were modestly attenuated after allowing for the competing risk of death (Table 4). Age remained significant in the participants with RVSP >30 mmHg but not the participants with RVSP >40 mmHg. The effect of NT-proBNP was attenuated with time.

Limiting the denominator to the 445 participants with an echocardiogram during follow-up, incidence rates (95% CI) for the first diagnosis of PH at eRVSPs >30 and >40 mmHg were 52.3 (43.5–62.4) and 26.3 (20.5–33.2)/1000 person-years during 2351 and 2665 person-years of follow-up, respectively. During follow-up to the end of 2016, 18 (1.3%) of the whole type 2 diabetes cohort were hospitalized for/with PH, of whom 14 (83.3%) were also identified as having PH by NEDA during follow-up, and 1 had a prior hospitalization with PH and was excluded from the analysis of new onset PH. We could not access medical records to confirm the ICD-10-AM coding for the three cases not captured with PH during follow-up by NEDA and, to be conservative, have not included them in the numerator.

## 4. Discussion

The present study provides novel data relating to the prevalence and incidence of echocardiographically detected PH in type 2 diabetes. Approaching a fifth of the FDS2 cohort had undergone echocardiography as part of usual care before entry and, of these, approximately one in eight had an eRVSP >40 mmHg. This increased to one in three for the lower 30 mmHg eRVSP threshold. The respective IRs were 8 and 14/1000 person-years. These prevalence and incidence data suggest that clinically significant PH is relatively common in type 2 diabetes. The present study has also identified expected and novel associates and determinants of PH that could assist in assessing risk and thus the need for investigations including echocardiography.

The most appropriate comparator for the present PH prevalence estimates in type 2 diabetes is the Armadale study, which utilized NEDA data to estimate the general population prevalence in an urban WA community geographically close to the FDS2 catchment area [8]. This latter study found that 9.1% (95% CI 8.6–9.7%) of the 10,314 who had an echocardiogram between 2003 and 2009 had an eRVSP >40 mmHg compared with 12.7% (9.1–17.4%) in the present study. Within the limitations of comparing the FDS2 and Armadale samples given minor geographical and temporal differences, albeit with data from the same echocardiographic database, the non-overlapping 95% CIs suggest that type 2 diabetes is associated with a 40% increased risk of PH. This may be a conservative estimate given that an Italian general population echocardiographic database study found a PH prevalence that was lower than in the Armadale study at 6.6% (6.2–6.9%) among 21,483 people [5]. Nevertheless, 9.4% and 19.0% of the whole Australian NEDA sample had an eRVSP >40 and >30 mmHg [8], respectively, again suggesting a 35–50% increase in the FDS2 cohort at both thresholds (12.7% and 32.7%, respectively, in the present study).

Our estimated minimum indicative prevalence of 2.6% for echocardiographically identified PH at an eRVSP of the >40 mmHg threshold in the total FDS2-recruited cohort with type 2 diabetes would fall to 0.9% if people identified with type 2 diabetes but not recruited were added to the denominator. This percentage would further reduce to 0.7% for the estimated 5586 people with confirmed type 2 diabetes in the catchment area during the FDS2 enrolment based on the total numbers from national databases and other sources [23]. The equivalent figure for the 165,450 people in the Armadale study population was similar at 0.6%. It is, however, difficult to interpret these data since the percentages are small and there are disparities (such as 19.2% of FDS2 participants versus only 6.2% of the Armadale population with echocardiographic data) that complicate comparisons.

Community-based echocardiographic screening studies involving people with diabetes have produced inconsistent results. Relative to FDS2 and the Armadale study, the Rotterdam study found a low overall prevalence of 2.6% with no significantly increased risk in people with diabetes of unspecified type [6]. Since the Rotterdam study was enriched for older individuals, the low prevalence and lack of an association with diabetes may have reflected a survivor effect. The Jackson Heart Study showed a 43% increased risk of PH in the almost 25% of its African American cohort who had diabetes [4], and a US veterans study found that the overall prevalence of diabetes of unspecified type in those with PH was 45% greater than that in the Veterans Health Affairs system as a whole (36% versus 25%) [24]. The results of these two studies align with the comparison of the present study and Armadale datasets.

We included the 30 mmHg eRVSP threshold because emerging evidence suggests that this lower cut-point has prognostic implications, including for death [8,25]. Using an eRVSP >30 mmHg would, based on our data, at least double PH prevalence in people with type 2 diabetes. Whether lowering the eRVSP threshold would prompt cost-effective investigations and treatment for PH sub-types is unknown. For example, there are increasing numbers of therapies that can improve exercise tolerance, delay clinical progression and, in some cases, reduce mortality in PAH [1], but these have been validated for people with an eRVSP >25 mmHg on RHC (equivalent to >40 mmHg by echocardiography).

There are no published general population or diabetes-specific incidence data for echocardiographically detected PH with which to compare the rates in FDS2 participants. Annual echocardiography is recommended in systemic sclerosis, a disease in which PAH has an annual incidence of 0.6–1.4% confirmed by RHC after echocardiography [26,27]. Although the patients with systemic sclerosis were an average of 10 years younger than those in the present study [26,27] and the incidence data were for PAH rather than the full PH spectrum, the annual incidence of PH in the present study (0.8%) further strengthens the case for the consideration of PH in the routine assessment of people with type 2 diabetes. The incidence of congestive cardiac failure in type 2 diabetes has been reported as high as 30.9 cases/1000 person-years in a large administrative database study [28], but our estimate of 7.6/1000 person-years for PH at the 40 mmHg eRVSP threshold is similar to rates of heart failure complicating diabetes found in other studies [29,30]. The high incidence of heart failure has prompted a push for early recognition and aggressive treatment of modifiable risk factors [28], and there could be a similar argument for this strategy in the case of PH.

Although the classification of PH into etiologic sub-groups was beyond the scope of the present study, a range of readily accessible independent variables were associated with prevalent and incident PH. An increase in plasma NT-proBNP was independently associated with PH in all multivariable analyses. Its strong predictive value in incident PH in the present study support its suggested use as a screening tool [31]. Since attenuation of the relationship with incident PH was observed only in the competing risk model, people with higher NT-proBNP may be dying before developing PH, thus weakening the prognostic relationship with incident PH. HsCRP was also a strong independent predictor of incident PH and, together with NT-proBNP, also predicts adverse outcomes from PH [32]. The only diabetes–specific risk factor was duration of disease with an increase of 3–4%/year in incident PH for each year increase in duration, regardless of eRVSP threshold. Other demographic (age and sex) and disease-related risk factors (cardiopulmonary conditions) were consistent with previous epidemiologic studies [4,5,6,7,8] and/or recognised etiologic factors [2]. This latter group includes left heart disease [14] and cardiac valvular disease [33], even if the only independent association was with prevalent PH at an RVSP >30 mmHg. The use of aspirin (prevalent PH) and lipid-modifying agents (incident PH) likely represent confounding by indication since these medications are indicated for people with, or at high risk of, cardiovascular disease.

The present study had limitations. There was potential selection bias in that FDS2 participants who had echocardiography may have had this investigation as part of assessment of cardiac conditions associated with PH, as with similar studies utilising databases [5,7,8]. We also assumed that patients who did not undergo echocardiography did not have PH but, if PH were present in some of these individuals, the overall prevalence/incidence would have been even higher. In addition, participants who did not have a measurable tricuspid regurgitation velocity may have been misclassified as not having PH, leading to further underestimation of prevalence/incidence. For these reasons, the present study represents the minimum indicative prevalence/incidence of PH in type 2 diabetes. The eRVSP was classified as unmeasurable/unmeasured in 41% of those who had echocardiographs compared with 32% in the Armadale study which also utilized the NEDA database [7]. However, >50% of the FDS2 participants were in the obese BMI range and this may have contributed to the higher rate in the present study through associated technical issues with obtaining echocardiographic variables [34], while participants in whom TRV was too small to measure or absent are typically included in analyses as they are very likely to have normal pulmonary pressures [6]. There was also a lack of data linkage between echocardiography and confirmatory RHC, while classification of PH sub–types and assessment of the relationship between PH and other echocardiographic parameters (see Supplementary Table S3) were beyond the scope of the present analyses. The strengths of our study are the large, well-characterized, community-based FDS2 participants with type 2 diabetes with a long duration of follow-up.

## 5. Conclusions

This is the first study to report the prevalence, incidence and associates of PH complicating type 2 diabetes in the community. Based on contemporary local comparative general population data and consistent with limited available data from previous studies, there is reasonable evidence that PH prevalence is increased by up to 50% in type 2 diabetes, while the incidence is similar to that in conditions with a high risk of PH such as systemic sclerosis and to the risk of heart failure complicating type 2 diabetes. The presence of PH should thus be considered as part of regular clinical assessment of individuals with type 2 diabetes. In addition to easily accessible demographic and clinical variables as risk factors, the present study adds weight to the potential of biomarkers such as NT-proBNP and hsCRP in predicting the emergence of PH in people with type 2 diabetes.

## Figures and Tables

**Table 1:** Baseline characteristics of type 2 diabetes participants by prevalent pulmonary hypertension (PH) status. Data are percentages, mean ± SD, geometric mean (SD range) or median {inter-quartile range}.

		RVSP > 30 mmHg			RVSP > 40 mmHg		
	All	No PH	Prevalent PH	*p*-Value	No PH	Prevalent PH	*p*-Value
Number (%)	1430 (100)	1338 (93.6)	92 (6.4)		1393 (97.4)	37 (2.6)	
Age (years)	65.5 ± 11.6	65.1 ± 11.6	71.2 ± 9.9	<0.001	65.3 ± 11.6	72.9 ± 8.2	<0.001
Sex (% male)	51.5	51.5	52.2	0.915	51.3	62.2	0.243
Ethnic background (%): Anglo-Celt	52.4	52.4	53.3		52.7	43.2	
Southern European	12.9	12.9	13.0		12.8	18.9	
Other European	7.1	7.3	4.3	0.558	7.3	2.7	0.259
Asian	4.3	4.3	4.3		4.4	2.7	
Aboriginal	7.5	7.2	12.0		7.3	16.2	
Mixed/other	15.7	15.8	13.0		15.6	16.2	
Currently married/*de facto* relationship (%)	62.6	63.2	54.3	0.096	62.8	54.1	0.303
Educated beyond primary level (%)	86.6	86.9	83.0	0.330	86.9	76.5	0.119
Smoking status (%): Never	42.4	42.4	42.4		42.2	48.6	
Ex-	46.7	46.6	47.8	0.963	46.8	43.2	0.765
Current	10.9	11.0	9.8		11.0	8.1	
Alcohol consumption (standard drinks/day)	0.1 {0–1.2}	0.1 {0–1.2}	0.1 {0–0.8}	0.015	0.1 {0–1.2}	0 {0–0.3}	0.018
Age at diabetes diagnosis (years)	55.6 ± 12.2	55.4 ± 12.3	59.1 ± 10.7	0.002	55.6 ± 12.3	58.8 ± 11.2	0.112
Diabetes duration (years)	8.0 {2.5–15.4}	8.0 {2.3–15.2}	13.0 {5.0–17.0}	0.002	8.0 {2.3–15.3}	15.0 {6.5–18.7}	0.003
Diabetes treatment (%): Diet	24.9	25.3	19.6		25.3	10.8	
Oral agents/non-insulin injectables	54.0	53.7	58.7	0.127	53.8	62.2	0.011
Insulin only	5.4	5.1	9.8		5.1	16.2	
Insulin/other agents	15.7	15.9	12.0		15.8	10.8	
Fasting serum glucose (mmol/L)	7.1 {6.1–8.8}	7.2 {6.2–8.9}	6.9 {5.9–8.2}	0.091	7.1 {6.2–8.9}	6.7 {5.5–7.9}	0.037
HbA_1c_ (%)	6.8 {6.2–7.7}	6.8 {6.2–7.7}	6.8 {6.2–7.4}	0.243	6.8 {6.2–7.7}	6.9 {6.3–7.7}	0.867
HbA_1c_ (mmol/mol)	51 {44–61}	51 {44–61}	51 {44–57}	0.243	51 {44–61}	52 {44–61}	0.867
A body shape index (m^11/6^/kg^2/3^)	0.081 ± 0.005	0.081 ± 0.005	0.082 ± 0.005	0.160	0.081 ± 0.005	0.084 ± 0.006	0.006
BMI (kg/m^2^)	31.3 ± 6.1	31.3 ± 6.1	31.1 ± 6.0	0.062	31.3 ± 6.1	30.1 ± 5.0	0.260
Heart rate (bpm)	70 ± 12	70 ± 12	73 ± 15	0.062	70 ± 12	73 ± 15	0.105
Supine systolic blood pressure (mmHg)	146 ± 22	145 ± 22	148 ± 23	0.296	146 ± 22	147 ± 23	0.643
Supine diastolic blood pressure (mmHg)	80 ± 12	80 ± 12	79 ± 16	0.308	80 ± 12	79 ± 17	0.593
Pulse pressure (mm Hg)	65 ± 18	65 ± 18	69 ± 20	0.030	65 ± 18	68 ± 20	0.355
Orthostatic hypotension (%)	31.8	30.7	48.2	0.001	31.5	45.5	0.092
Antihypertensive medication (%)	74.0	73.1	87.0	0.003	73.5	91.9	0.012
Total serum cholesterol (mmol/L)	4.4 ± 1.1	4.4 ± 1.1	4.0 ± 1.2	0.001	4.4 ± 1.1	3.9 ± 1.1	0.005
Serum HDL-cholesterol (mmol/L)	1.23 ± 0.34	1.23 ± 0.34	1.17 ± 0.30	0.059	1.23 ± 0.34	1.14 ± 0.32	0.095
Serum triglycerides (mmol/L)	1.5 (0.9–2.6)	1.5 (0.9–2.6)	1.4 (0.9–2.4)	0.251	1.5 (0.9–2.6)	1.5 (0.9–2.5)	0.800
Lipid-modifying medication (%)	68.7	68.4	72.8	0.417	68.4	81.1	0.109
Aspirin therapy (%)	37.5	36.4	54.3	0.001	37.2	48.6	0.171
Plasma NTpro–BNP (pg/mL)	79 (18–344)	72 (18–289)	357 (69–1856)	<0.001	75 (18–310)	651 (119–3572)	<0.001
Serum hsCRP (mg/L)	2.5 (0.8–7.7)	2.5 (0.8–7.5)	3.1 (0.9–10.7)	0.066	2.5 (0.8–7.6)	3.4 (0.9–13.2)	0.160
Atrial fibrillation (% on ECG coding)	4.5	3.5	18.9	<0.001	3.8	27.8	<0.001
Left ventricular hypertrophy (%)	2.0	1.9	3.3	0.418	1.9	5.6	0.158
Cerebrovascular disease (%)	8.5	7.5	22.8	<0.001	7.8	32.4	<0.001
Coronary heart disease (%)	28.7	26.9	55.4	<0.001	28.0	56.8	<0.001
History of heart failure (%)	6.3	4.2	37.0	<0.001	4.2	48.6	<0.001
Peripheral arterial disease (%)	22.5	21.9	30.8	0.068	22.3	29.7	0.317
Peripheral sensory neuropathy (%)	58.2	57.2	73.6	0.002	57.8	75.7	0.029
eGFR category (%): ≥90 mL/min/1.73 m^2^	38.7	40.0	17.6		39.2	16.7	
60–89 mL/min/1.73 m^2^	44.9	44.5	50.5		44.9	47.2	
45–59 mL/min/1.73 m^2^	8.8	8.7	9.9	<0.001	8.9	5.6	<0.001
30–44 mL/min/1.73 m^2^	4.8	4.6	7.7		4.8	8.3	
<30 mL/min/1.73 m^2^	2.8	2.0	14.3		2.3	22.2	
Urinary albumin:creatinine ratio (mg/mmol)	3.3 (0.8–12.6)	3.1 (0.8–11.6)	6.2 (1.1–36.9)	0.001	3.2 (0.8–11.7)	12.2 (1.5–100.7)	0.001
History of ESKD (%)	0.8	0.4	6.5	<0.001	0.6	10.8	<0.001
Any diabetic retinopathy (%)	36.4	36.2	38.8	0.643	36.3	39.4	0.717
History of COPD (%)	2.5	1.8	13.0	<0.001	1.9	24.3	<0.001
Charlson Comorbidity Index (%): 0	75.5	78.0	38.0		76.8	21.6	
1 or 2	16.7	15.7	31.5	<0.001	16.2	35.1	<0.001
≥3	7.9	6.4	30.4		7.0	43.2	
History of cardiac valvular disease (%)	2.4	1.5	16.3	<0.001	2.0	18.9	<0.001
History of sleep apnoea (%)	4.1	3.7	8.7	0.029	3.8	13.5	0.015

**Table 2:** Independent associates of prevalent and incident pulmonary hypertension (PH). Odds ratios (OR) and 95% CI are shown for prevalent PH and cause-specific hazard ratios (csHR) and 95% CI for incident PH.

	Prevalent PH (RVSP > 30 mmHg)		Prevalent PH (RVSP > 40 mmHg)		Incident PH (RVSP > 30 mmHg)		Incident PH (RVSP > 40 mmHg)	
	OR (95% CI)	*p*-value	OR (95% CI)	*p*-value	csHR (95% CI)	*p*-value	csHR (95% CI)	*p*-value
Age (increase of 1 year)					1.03 (1.01, 1.05)	0.001	1.03 (1.002, 1.05)	0.035
Male					0.67 (0.47, 0.97)	0.036		
Married/*de facto*							0.57 (0.36, 0.92)	0.022
Diabetes duration (1 year increase)					1.03 (1.01, 1.05)	0.002	1.04 (1.01, 1.06)	0.003
Heart rate (increase of 1 bpm)							1.02 (1.001, 1.04)	0.037
Orthostatic hypotension	1.87 (1.15, 3.02)	0.011						
On lipid-modifying medication							3.46 (1.68, 7.09)	0.001
Aspirin use	1.65 (1.03, 2.65)	0.038						
Ln(hsCRP (mg/L)) ^a^					1.43 (1.21, 1.68)	<0.001	1.55 (1.25, 1.93)	<0.001
Ln(NT-proBNP (pg/mL)) ^a^	1.53 (1.30, 1.79)	<0.001	1.88 (1.50, 2.35)	<0.001	1.61 (1.41, 1.84)	<0.001	1.72 (1.46, 2.02)	<0.001
History of heart failure	5.71 (3.20, 10.19)	<0.001	4.56 (2.03, 10.22)	<0.001				
History of valvular disease	4.22 (1.81, 9.83)	0.001						
History of COPD			5.56 (2.01, 15.35)	0.001				

^a^ A 2.72-fold increase in variable x corresponds to an increase of 1 in ln(variable x).

**Table 3:** Baseline characteristics of type 2 diabetes participants without prevalent pulmonary hypertension (PH) by incident PH status to end of December 2016. Data are percentages, mean ± SD, geometric mean (SD range) or median {interquartile range}.

	RVSP > 30 mmHg			RVSP > 40 mmHg		
	No PH	Incident PH	*p*-Value	No PH	Incident PH	*p*-Value
Number (%)	1215 (90.8)	123 (9.2)		1323 (95.0)	70 (5.0)	
Age (years)	64.5 ± 11.5	70.9 ± 10.7	<0.001	64.9 ± 11.5	72.0±10.4	<0.001
Sex (% male)	52.8	38.2	0.002	51.9	40.0	0.065
Ethnic background (%): Anglo–Celt	51.6	60.2		52.2	61.4	
Southern European	13.0	12.2		13.0	8.6	
Other European	7.6	4.9	0.394	7.3	5.7	0.483
Asian	4.4	4.1		4.4	4.3	
Aboriginal	7.1	8.1		7.1	10.0	
Mixed/other	16.4	10.6		15.9	10.0	
Currently married/*de facto* relationship (%)	64.0	55.3	0.063	63.7	45.7	0.003
Educated beyond primary level (%)	86.9	86.2	0.775	86.6	92.3	0.257
Smoking status (%): Never	42.4	42.3		42.5	37.1	
Ex–	46.8	44.7	0.707	46.7	48.6	0.514
Current	10.8	13.0		10.8	14.3	
Alcohol consumption (standard drinks/day)	0.1 {0–1.2}	0.1 {0–1.2}	0.054	0.1 {0–1.2}	0.1 {0–0.9}	0.208
Age at diabetes diagnosis (years)	55.2 ± 12.2	57.7 ± 13.1	0.028	55.4 ± 12.2	57.9 ± 12.7	0.120
Diabetes duration (years)	7.0 {2.0–15.0}	12.3 {6.0–19.0}	<0.001	8.0 {2.0–15.0}	15.1 {7.5–20.7}	<0.001
Diabetes treatment (%): Diet	25.7	21.1		25.8	15.7	
Oral agents/non-insulin injectables	54.0	51.2	0.137	53.7	55.7	0.151
Insulin only	4.7	8.9		5.0	7.1	
Insulin/other agents	15.7	18.7		15.5	21.4	
Fasting serum glucose (mmol/L)	7.1 {6.1–8.8}	7.3 {6.2–9.0}	0.705	7.1 {6.1–8.8}	7.8 {6.3–9.0}	0.238
HbA_1c_ (%)	6.8 {6.2–7.7}	6.7 {6.3–7.7}	0.887	6.8 {6.2–7.7}	6.8 {6.3–7.5}	0.631
HbA_1c_ (mmol/mol)	51 {44–61}	50 {45–61}	0.887	51 {44–61}	51 {45–61}	0.631
A body shape index (m^11/6^/kg^2/3^)	0.081 ± 0.005	0.082 ± 0.006	0.064	0.081 ± 0.005	0.082 ± 0.006	0.127
BMI (kg/m^2^) (%):	31.2 ± 6.0	31.6 ± 6.7	0.500	31.3 ± 6.0	31.8 ± 7.0	0.440
Heart rate (bpm)	69 ± 12	72 ± 14	0.114	70 ± 12	73 ± 15	0.051
Systolic blood pressure (mmHg)	145 ± 21	151 ± 27	0.012	145 ± 21	151 ± 32	0.135
Diastolic blood pressure (mmHg)	80 ± 12	79 ± 13	0.157	80 ± 12	78 ± 15	0.299
Pulse pressure (mm Hg)	64 ± 17	72 ± 21	<0.001	65 ± 17	73 ± 24	0.011
Orthostatic hypotension (%)	29.7	40.8	0.017	31.2	36.4	0.415
Antihypertensive medication (%)	71.9	84.4	0.003	72.9	85.5	0.024
Total serum cholesterol (mmol/L)	4.4 ± 1.1	4.4 ± 1.1	0.851	4.4 ± 1.1	4.1 ± 0.9	0.061
Serum HDL–cholesterol (mmol/L).4294	1.23 ± 0.33	1.30 ± 0.41	0.050	1.23 ± 0.34	1.26 ± 0.36	0.505
Serum triglycerides (mmol/L)	1.5 (0.9–2.6)	1.6 (1.0–2.6)	0.371	1.5 (0.9–2.6)	1.5 (0.9–2.3)	0.585
Lipid-modifying medication (%)	67.7	75.4	0.083	67.4	87.0	<0.001
Aspirin therapy (%)	35.5	45.5	0.037	36.4	52.9	0.007
Plasma NTpro–BNP (pg/mL)	64 (17–246)	209 (48–909)	<0.001	70 (18–274)	306 (62–1507)	<0.001
Serum hsCRP (mg/L)	2.4 (0.8–7.3)	3.4 (1.2–9.9)	0.001	2.4 (0.8–7.3)	3.9 (1.2–13.0)	<0.001
Atrial fibrillation (% on ECG coding)	2.9	8.9	0.002	3.3	14.3	<0.001
Left ventricular hypertrophy (%)	1.7	4.1	0.075	1.6	7.1	0.008
Cerebrovascular disease (%)	6.7	14.6	0.003	7.5	14.3	0.062
Coronary heart disease (%)	25.7	39.0	0.003	27.1	45.7	0.001
History of heart failure (%)	3.7	8.9	0.014	4.5	18.6	<0.001
Peripheral arterial disease (%)	20.8	33.3	0.002	21.4	38.6	0.002
Peripheral sensory neuropathy (%)	56.4	65.0	0.069	57.0	71.4	0.018
eGFR category (%): ≥90 mL/min/1.73m^2^	42.1	20.3		40.5	15.7	
60–89 mL/min/1.73m^2^	44.1	48.8		44.5	51.4	
45–59 mL/min/1.73m^2^	8.3	13.0	<0.001	8.8	10.0	<0.001
30–44 mL/min/1.73m^2^	3.9	12.2		4.2	14.3	
<30 mL/min/1.73m^2^	1.7	5.7		2.0	8.6	
Urinary albumin:creatinine ratio (mg/mmol)	3.0 (0.8–10.9)	4.8 (1.1–20.0)	<0.001	3.0 (0.8–11.1)	6.4 (1.5–27.7)	<0.001
History of ESKD (%)	0.2	2.4	0.012	0.5	2.9	0.057
Any diabetic retinopathy (%)	35.1	47.1	0.010	35.7	47.8	0.053
History of COPD (%)	1.8	1.6	>0.999	1.9	2.9	0.643
Charlson Comorbidity Index (%): 0	79.6	61.8		78.1	52.9	
1 or 2	15.1	22.0	<0.001	15.6	27.1	<0.001
≥3	5.3	16.3		6.3	20.0	
History of valve disease (%)	1.4	2.4	0.420	1.7	7.1	0.011
History of sleep apnoea (%)	3.8	3.3	>0.999	3.9	1.4	0.516

**Table 4:** Fine and Gray competing risk models of independent determinants of incident pulmonary hypertension (PH). Subdistribution hazard ratios (sdHR) and 95% confidence intervals (CI) are shown.

	Incident PH (RVSP > 30 mmHg)		Incident PH (RVSP > 40 mmHg)	
	sdHR (95% CI)	*p*-value	sdHR (95% CI)	*p*-value
Main:				
Age (increase of 1 year)	1.03 (1.01, 1.05)	0.005		
Male	0.63 (0.43, 0.91)	0.015		
Married/*de facto*			0.58 (0.36, 0.95)	0.030
Diabetes duration (increase of 1 year)	1.03 (1.01, 1.04)	0.010	1.03 (1.01, 1.06)	0.013
Heart rate (increase of 1 bpm)				
On lipid-modifying medication			3.58 (1.78, 7.18)	<0.001
Ln(NT-proBNP (pg/mL)) ^a^	1.55 (1.35, 1.79)	<0.001	1.78 (1.52, 2.09)	<0.001
Ln(hsCRP (mg/L)) ^a^	1.33 (1.13, 1.58)	0.001	1.42 (1.14, 1.76)	0.002
Time-varying:				
Ln(NT-proBNP (pg/mL)) * ln(time)	0.92 (0.84, 0.999)	0.047	0.90 (0.82, 0.99)	0.035

^a^ A 2.72-fold increase in x corresponds to an increase of 1 in * ln(x).

## Data Availability

The datasets generated during this study and/or as a result of analysis are available from the corresponding author on reasonable request.

## Supplementary Materials

The following are available online at https://www.mdpi.com/article/10.3390/jcm10194503/s1, Table S1: Baseline characteristics of type 2 diabetes participants by (i) NEDA status, and (ii) measured/measurable RVSP status. Data are percentages, mean ± SD, geometric mean (SD range) or median [inter-quartile range]. Table S2: Independent associates of (i) capture by NEDA database, and (ii) RVSP measured in FDS2 participants with confirmed type 2 diabetes. Table S3: Echocardiographic parameters for participants with a matched echocardiographic study by prevalent and incident PH at time PH was first identified by echocardiography. Data are presented as number (n), percentages (%), mean ± SD or median [inter-quartile range]. The number of measurements (n) per variable is presented when it differs from the total number. LVDD = left ventricular diastolic dimension, LV = left ventricular, LVEF = left ventricular ejection fraction, LA = left atrial, eRVSP = estimated right ventricular systolic pressure and TR = tricuspid regurgitation. Aortic stenosis (>mild) was defined as aortic valve mean gradient >20 mmHg, Mitral stenosis (>mild) was defined as mitral valve mean gradient >5 mmHg. Left heart disease (any manifestation) represents the presence of any one or more of the following: LVEF < 55%, >mild mitral stenosis or regurgitation, >mild aortic stenosis or regurgitation, LA volume index > 34 mL/m^2^ or E:e’ ratio > 15.
