# Supplementary material for: Prevalence, Incidence and Associates of Pulmonary Hypertension Complicating Type 2 Diabetes: Insights from the Fremantle Diabetes Study Phase 2 and National Echocardiographic Database of Australia
Source: J Clin Med. 2021 Sep 29;10(19):4503. doi: 10.3390/jcm10194503 (PMC8509549; doi:10.3390/jcm10194503)
Supplement: Supplementary file 1 [file jcm-10-04503-s001.zip › jcm-1381733-supplementary.pdf]

Table S1. Baseline characteristics of type 2 diabetes participants by i) NEDA status, and ii) measured/measurable RVSP status. Data are percentages, mean ± SD, geometric mean (SD range) or median [inter-quartile range].

	All	Not in NEDA	In NEDA	*P*-value	No measured/ measurable RVSP	Measured RVSP	*P*-value
Number (%)	1430 (100)	827 (57.8)	603 (42.2)		242 (40.1)	361 (59.9)	
Age (years)	65.5±11.6	64.0±11.7	67.5±11.0	<0.001	64.1±11.1	69.9±10.4	<0.001
Sex (% male)	51.5	51.0	52.2	0.668	60.7	46.5	0.001
APOE4 genotype (%)	23.5	24.9	21.7	0.183	21.5	21.8	>0.999
Ethnic background (%):				0.059			0.147
Anglo-Celt	52.4	50.3	55.4		54.5	56.0	
Southern European	12.9	13.3	12.4		11.6	13.0	
Other European	7.1	6.8	7.6		10.7	5.5	
Asian	4.3	4.8	3.6		2.1	4.7	
Aboriginal	7.5	6.9	8.3		12.4	12.7	
Mixed/other	15.7	17.9	12.6		8.7	8.0	
Not fluent in English (%)	10.8	10.8	10.9	0.931	10.3	11.4	0.790
Currently married/*de facto* relationship (%)	62.6	66.0	57.9	0.002	60.3	56.2	0.355
Educated beyond primary level (%)	86.6	87.2	85.7	0.426	83.8	87.1	0.278
Smoking status (%)				0.272			0.038
Never	42.4	43.8	40.4		34.7	44.2	
Ex-	46.7	46.2	47.4		50.2	45.6	
Current	10.9	10.0	12.2		15.1	10.3	
TV viewing >21 hours/week (%)	34.2	34.2	34.2	>0.999	34.2	34.1	>0.999
Alcohol consumption (standard drinks/day)	0.1 [0-1.2]	0.1 [0-1.2]	0.1 [0-1.2]	0.459	0.3 [0-1.2]	0.1 [0-1.2]	0.058
Age at diabetes diagnosis (years)	55.6±12.2	55.5±12.0	55.8±12.6	0.600	52.7±12.7	57.9±12.6	<0.001
Diabetes duration (years)	8.0 [2.5-15.4]	6.0 [2.0-13.8]	11.0 [3.7-17.3]	<0.001	10.0 [3.0-17.2]	11.9 [4.0-17.8]	0.308
Diabetes treatment (%)				0.001			0.003
Diet	24.9	26.9	22.2		20.2	23.5	
Oral hypoglycemic agents (OHAs)/non-insulin injectables	54.0	55.2	52.4		46.7	56.2	
Insulin only (%)	5.4	3.8	7.6		8.7	6.9	

Insulin+OHAs/non-unsulin injectables	15.7	14.2	17.7		24.4	13.3	
Fasting serum glucose (mmol/L)	7.1 [6.1-8.8]	7.2 [6.2-8.7]	7.0 [6.0-8.9]	0.190	7.4 [6.1-9.1]	6.9 [5.9-8.7]	0.050
HbA_{1c} (%)	6.8 [6.2-7.7]	6.8 [6.2-7.7]	6.8 [6.2-7.7]	0.832	7.0 [6.3-8.0]	6.7 [6.2-7.4]	0.002
HbA_{1c} (mmol/mol)	51 [44-61]	51 [44-61]	51 [44-61]	0.832	53 [45-64]	50 [44-57]	0.002
ABSI ($m^{11/6}/kg^{2/3}$)	0.081±0.005	0.081±0.005	0.082±0.005	<0.001	0.082±0.005	0.082±0.006	0.819
BMI (kg/m^2)	31.3±6.1	30.9±5.7	31.8±6.4	0.007	33.2±6.6	30.8±6.1	<0.001
Central obesity (by waist circumference; %)	71.3	69.1	74.2	0.043	77.6	71.9	0.128
Heart rate (bpm)	70±12	70±12	70±13	0.472	70±12	71±14	0.389
Supine systolic blood pressure (mmHg)	146±22	144±21	148±23	0.002	148±22	148±23	0.895
Supine diastolic blood pressure (mmHg)	80±12	80±11	80±13	0.401	81±14	79±13	0.020
Pulse pressure (mm Hg)	65±18	64±17	68±19	<0.001	66±17	69±20	0.068
Orthostatic hypotension (%)	31.8	27.9	37.2	<0.001	36.0	38.0	0.664
Antihypertensive medication (%)	74.0	68.3	81.7	<0.001	79.7	83.1	0.333
Total serum cholesterol (mmol/L)	4.4±1.1	4.4±1.1	4.2±1.1	0.003	4.3±1.1	4.2±1.1	0.658
Serum HDL-cholesterol (mmol/L)	1.23±0.34	1.22±0.32	1.25±0.36	0.137	1.21±0.30	1.27±0.39	0.051
Serum triglycerides (mmol/L)	1.5 (0.9-2.6)	1.6 (0.9-2.6)	1.5 (0.9-2.6)	0.369	1.6 (0.9-2.8)	1.5 (0.9-2.4)	0.100
Lipid-modifying medication (%)	68.7	65.0	73.7	0.001	73.9	73.6	>0.999
Aspirin therapy (%)	37.5	31.6	45.7	<0.001	44.0	46.8	0.505
NTpro-BNP (pg/mL)	79 (18-344)	55 (14-212)	130 (29-576)	<0.001	86 (21-344)	171 (38-764)	<0.001
hsCRP (mg/L)	2.5 (0.8-7.7)	2.4 (0.8-7.2)	2.7 (0.8-8.4)	0.083	2.8 (0.9-8.4)	2.6 (0.8-8.4)	0.404
Atrial fibrillation (% on ECG coding)	4.5	3.1	6.4	0.004	2.5	9.0	0.002
Left ventricular hypertrophy (%)	2.0	1.5	2.7	0.122	2.5	2.8	>0.999
Cerebrovascular disease (%)	8.5	5.7	12.3	<0.001	9.9	13.9	0.165
Coronary heart disease (%)	28.7	19.2	41.8	<0.001	39.3	43.5	0.313
History of heart failure (%)	6.3	2.7	11.3	<0.001	6.6	14.4	0.004
Peripheral arterial disease (%)	22.5	21.0	24.5	0.139	20.7	26.9	0.100
Peripheral sensory neuropathy (%)	58.2	55.1	62.6	0.005	58.1	65.6	0.071
eGFR category (%)				<0.001			0.001
≥90 mL/min/1.73m^2	38.7	45.6	29.1		36.1	24.4	
60-89 mL/min/1.73m^2	44.9	42.0	48.9		47.7	49.7	
45-59 mL/min/1.73m^2	8.8	7.5	10.5		10.0	10.8	
30-44 mL/min/1.73m^2	4.8	3.6	6.5		2.5	9.2	
<30 mL/min/1.73m^2	2.8	1.2	5.0		3.7	5.8	

Urinary albumin:creatinine ratio (mg/mmol)	3.3 (0.8-12.6)	2.7 (0.8-9.2)	4.1 (0.9-18.6)	<0.001	3.8 (0.8-17.9)	4.4 (1.0-19.0)	0.237
History of ESKD (%)	0.8	0.2	1.7	0.006	0.4	2.5	0.056
Any diabetic retinopathy (%)	36.4	34.5	38.9	0.092	38.1	39.5	0.797
History of COPD (%)	2.5	1.1	4.5	<0.001	4.5	4.4	>0.999
Charlson Comorbidity Index[a] (%):				<0.001			0.022
0	75.5	82.3	65.8		70.7	62.6	
1 or 2	16.7	13.5	21.1		20.7	21.3	
≥3	7.9	4.1	13.1		8.7	16.1	
History of valve disease (%)	2.4	1.3	4.0	0.002	1.7	5.5	0.018
History of sleep apnoea (%)	4.1	3.1	5.3	0.043	6.6	4.4	0.269

[a]In the past 5 years and excluding diabetes and diabetes-specific complications

Table S2. Independent associates of i) capture by NEDA database, and ii) RVSP measured in FDS2 participants with confirmed type 2 diabetes.

	NEDA no/yes		RVSP measured	
	OR (95% CI)	*P*-value	OR (95% CI)	*P*-value
Age (increase of 1 year)			1.04 (1.02, 1.06)	<0.001
Male			0.49 (0.34, 0.71)	<0.001
Other European			0.41 (0.21, 0.80)	0.009
Mixed/other ethnic background	0.65 (0.47, 0.90)	0.010		
Married/*de facto*	0.76 (0.60, 0.96)	0.021		
BMI (increase of 1 kg/m^2)	1.04 (1.02, 1.06)	<0.001	0.95 (0.92, 0.98)	0.001
Age at diabetes diagnosis (increase of 1 year)	0.98 (0.97, 0.99)	<0.001		
Using insulin			0.50 (0.33, 0.75)	0.001
On aspirin	1.40 (1.10, 1.79)	0.007		
eGFR ≥ 90 $mL/min/1.73m^2$	0.64 (0.49, 0.83)	0.001		
eGFR = 30-44 $mL/min/1.73m^2$			2.95 (1.13, 7.71)	0.027
Ln(NT-proBNP (pg/mL))[a]	1.40 (1.27, 1.55)	<0.001		
Atrial fibrillation			3.13 (1.22, 8.01)	0.018
Coronary heart disease	2.03 (1.55, 2.66)	<0.001		
History of heart failure			2.41 (1.26, 4.58)	0.008
Charlson Comorbidity Index ≥ 3	1.89 (1.17, 3.06)	<0.001		

[a]A 2.72-fold increase in x corresponds to an increase of 1 in ln(x)

Table S3. Echocardiographic parameters for participants with a matched echocardiographic study by prevalent and incident PH at time PH was first identified by echocardiography. Data are presented as number (n), percentages (%), mean±SD or median [inter-quartile range]. The number of measurements (n) per variable is presented when it differs from the total number. LVDD = left ventricular diastolic dimension, LV= left ventricular, LVEF= left ventricular ejection fraction, LA= left atrial, eRVSP= estimated right ventricular systolic pressure and TR= tricuspid regurgitation. Aortic stenosis (>mild) was defined as aortic valve mean gradient > 20 mmHg, Mitral stenosis (>mild) was defined as mitral valve mean gradient > 5 mmHg. Left heart disease (any manifestation) represents the presence of any one or more of the following: LVEF <55%, > mild mitral stenosis or regurgitation, > mild aortic stenosis or regurgitation, LA volume index> 34 ml/m^2 or E:e' ratio >15.

	Prevalent PH		Incident PH	
	RVSP >30 mmHg	RVSP >40 mmHg	RVSP >30 mmHg	RVSP >40 mmHg
Number	90	35	123	70
Age (years)	68.8±9.7	70.7±8.4	74.5±10.7	75.5±10.6
Sex (% male)	52.2	62.9	39.0	41.4
LVDD (cm)	4.8±0.6 (n=56)	4.7±0.6 (n=17)	4.8±0.7 (n=81)	4.8±0.8 (n=43)
LV mass index (g/m^2)	103 [83-123] (n=48)	112 [82-130] (n=15)	96 [83-119] (n=56)	109 [90-125] (n=28)
LVEF (%)	64 [59-70] (n=57)	64 [60-72] (n=19)	60 [50-66] (n=103)	58 [38-63] (n=59)
Mitral inflow E velocity (cm/s)	91±27 (n=71)	102±31 (n=27)	104±32 (n=100)	118±34 (n=48)
Mitral inflow E:A ratio	0.89 [0.76-1.19] (n=64)	0.90 [0.73-1.33] (n=21)	1.04 [0.79-1.47] (n=88)	1.20 [0.88-1.84] (n=41)
Septal E:e' ratio	10 [8-12] (n=34)	13 [10-16] (n=10)	14 [10-20] (n=69)	21 [14-23] (n=32)
LA volume index (ml/m^2)	35 [26-43] (n=48)	35 [27-48] (n=15)	36 [25-47] (n=53)	47 [36-57] (n=27)
Non-sinus rhythm (%, including atrial fibrillation)	31.1	42.9	26.8	40.0
eRVSP (mmHg)	34 [36-41]	43 [41-49]	39 [34-46]	47 [43-53]
TR peak velocity (cm/s)	280 [270-300]	304 [300-328]	290 [268-320]	323 [310-348]
Left heart disease (any manifestation, %)	22.2	20.0	56.1	60.0
